# A retrospective study on cats with incompletely or marginally excised sarcomas treated with a bleomycin-only electrochemotherapy protocol: 66 cases

**DOI:** 10.3389/fvets.2026.1902206

**Published:** 2026-09-18

**Authors:** Kaitlan Hovis, Quincy Souquet, Shawn Ferguson, Jeffrey N. Bryan, Chelsea Tripp

**Affiliations:** 1 Bridge Animal Referral Center (BARC), Edmonds, WA, United States; 2 Department of Veterinary Medicine and Surgery, University of Missouri-Columbia, Columbia, MO, United States

**Keywords:** bleomycin, cat, electrochemotherapy, injection-site sarcomas, sarcoma

## Abstract

**Introduction:**

Electrochemotherapy (ECT) is a novel therapy used as an effective and safe treatment modality for both cats and dogs with various tumor types. This retrospective study aimed to evaluate the disease-free interval (DFI) for cats with incompletely excised or marginally excised sarcomas treated with a bleomycin-only ECT protocol.

**Methods:**

A single institution retrospective evaluation was performed to identify cats as having undergone surgical removal of their sarcoma tumor followed by ECT from March 2015 to July 2024. In the absence of a standardized grading system for feline sarcomas, cats were stratified into high-grade and low-grade groups with primary emphasis on mitotic count. Median DFI was subsequently evaluated for each group.

**Results:**

A total of 66 cats were identified and were divided into a high-grade group, which consisted of 43 cats treated with an average of 2 ECT treatments, and a low-grade group, which consisted of 23 cats treated with a single ECT treatment. Intravenous bleomycin was injected followed by a subcutaneous injection of bleomycin along the surgical scar in a 2:1 ratio. The surgical scar, as well as 2 cm of tissue surrounding the scar was then treated with electroporation with the following parameters: 8 biphasic waveforms; pulses duration of 100 μs; pulse repetition frequency of 1 Hz and pulse amplitude 1,200 volts/cm. Median DFI for the cats in the low-grade group was not reached whereas the median DFI for cats within the high-grade group was 389 days. This was statistically significant between the two groups.

**Conclusion:**

This is the first study to evaluate a bleomycin-only ECT protocol in cats. Bleomycin-only ECT improved DFI compared to surgery alone for cats with incompletely or marginally excised sarcomas.

## 1 Introduction

Sarcomas are mesenchymal neoplasms that have been recognized in cats for decades. Historically, there has not been a standardized grading system, although a grading system has recently been proposed (1), these tumors are commonly classified into two categories: non-injection site sarcomas (non-ISS) or spontaneous sarcomas and injection-site sarcomas (ISS). ISSs typically exhibit more aggressive biological behavior (2–4). Although treatment for sarcomas aims for wide surgical margins (5), tumor location frequently limits the feasibility of achieving adequate excision necessitating the use of additional therapy.

Adjuvant therapy, utilizing radiation therapy, has become standard of care for feline patients, with incompletely or marginally excised sarcoma tumors, to help decrease the potential for local disease recurrence (6–11). Radiation therapy can be challenging because of multiple anesthetic episodes, acute and chronic side effects (12), availability, and expense, making electrochemotherapy a desirable option for clients.

Electrochemotherapy (ECT) is a locoregional anticancer treatment that uses brief electric pulses to transiently increase cell membrane permeability, allowing enhanced uptake of chemotherapeutic agents, like bleomycin and cisplatin (13–15).

Previous studies have identified ECT as an effective and safe treatment option to delay recurrence of both feline and canine cutaneous and subcutaneous tumors, including mast cell tumors (16–18), melanomas (19, 20), carcinomas (21–28), and sarcomas (29–33). ECT has also been used in case reports to successfully treat feline progressive histiocytosis (34) and feline bilateral pleomorphic rhabdomyosarcoma (35). However, no studies, to date, describe using bleomycin both intravenously and intralesionally with electroporation for the treatment of feline sarcomas.

The primary goal of this study was to retrospectively determine disease-free interval (DFI) for client-owned cats with previously incompletely or marginally excised sarcomas using a bleomycin-only ECT protocol. A secondary goal was to describe electrochemotherapy side effects in treated cats.

## 2 Materials and methods

### 2.1 Patient selection and grouping

Medical records from Bridge Animal Referral Center, of client-owned cats treated with ECT as adjuvant post-surgical therapy for sarcomas, between March 2015 and July 2024 were retrospectively reviewed. Collected data included signalment, diagnosis, histopathological findings, date of surgery, number of ECT treatments, with all cats receiving bleomycin, both intralesionally and intravenously, side effects, additional chemotherapy post ECT, date of death, and follow-up records provided by BARC or referring veterinarians as well as any updates directly from clients.

In the absence of a standardized histologic grading system for feline sarcomas and the retrospective nature of the study, cases were stratified into high-grade and low-grade groups based primarily on mitotic count. Mitotic count was selected as the principal grading criterion because detailed assessments of other histologic features commonly used for tumor grading, such as necrosis and inflammation (1, 36), were inconsistently reported across pathology reports due to the involvement of multiple pathologists. The high-grade group included injection-site sarcomas, fibrosarcomas with a mitotic count ≥10, soft tissue sarcomas with a mitotic count ≥10, peripheral nerve sheath tumors with a mitotic count of ≥10, extraskeletal osteosarcomas, chondrosarcomas, and anaplastic sarcomas. Cases of subcutaneous hemangiosarcoma, those lacking a reported mitotic count, patients with gross disease at time of ECT, and those without documentation of the drugs used for ECT were excluded.

Tumors were considered incompletely excised if tumor cells were present at the margins of submitted tissue or if the margins were described as <1 mm. Marginally excised tumors included tumors that were removed with <3 cm of normal tissue surrounding the sarcoma.

### 2.2 Anesthesia protocol

All cats received an intravenous catheter. Sedation was provided using 0.2 mg/kg IV butorphanol (Dolorex, Merck, Madison, NJ, USA) and 0.1 mg/kg IV midazolam (Hospira Inc., Lake Forest, IL, USA). General anesthesia was induced using either intravenous alfaxolone (Alfaxan, Zoetis, Kalamazoo, MI, USA) at 2 mg/kg to effect or propofol (Hospira Inc., Lake Forest, IL, USA; Sagent, Schaumburg, IL, USA) at 6 mg/kg to effect. The choice of whether to use propofol or alfaxolone was determined by the attending clinician. Anesthesia was maintained with isoflurane gas (Isospore, Dechra, Overland Park, KS, USA).

### 2.3 Electrochemotherapy protocol and follow-up

Bleomycin (Northstar Rx LLC, Memphis, TN, USA) was injected intravenously. After 8 min, a local injection of bleomycin was administered into the surgical scar for a total bleomycin dose of 15 IU/m^2^. The intralesional bleomycin was injected into the subcutaneous space by advancing the needle through the skin, aspirating to confirm that no blood was present in the needle hub, and then injecting the bleomycin while withdrawing the needle along the tract. This was repeated to the entire length of the surgical scar. On average the total bleomycin dose was split between intravenous and intralesional administration in a 2:1 distribution, respectively, using an institutionally designed protocol. Following the intralesional injection of bleomycin, electric pulses were then immediately delivered to the surgical scar, as well as 2 cm of tissue surrounding the surgical scar. Electric pulses were provided via a veterinary-certified electroporator (Onkodisruptor®) with modified caliper electrodes: 8 biphasic waveforms; pulses duration of 100 μs; pulse repetition frequency of 1 Hz and pulse amplitude 1,200 volts/cm. The area to be treated was shaved and electroconductive gel was used to optimize electrode contact. Following treatment, the electroconductive gel was gently removed using gauze.

Patients were monitored throughout the procedure using pulse oximetry and cardiac monitoring. If a second ECT session was recommended, it was performed, on average, 2 weeks after the initial treatment. Following the conclusion of the ECT session(s), physical exams were recommended at a frequency of every 3–4 months over the first year following treatment to monitor for any evidence of local recurrence.

### 2.4 Side effects

Side effects of ECT were evaluated following each ECT treatment and were graded using the Veterinary Cooperative Oncology Group—Common Terminology Criteria for Adverse Events (VCOG-CTCAEv2) (37).

### 2.5 Statistics

DFI was calculated from the date of surgery until progressive disease was documented (local or metastatic). Curves for DFI for both groups of cats were performed using the Kaplan–Meier Survival Analysis with a 95% confidence interval. Statistical analysis was performed using SPSS software (version 10.00 SPSS; Chicago, IL, USA). Data was censored if cats were lost to follow-up, died of disease outside of sarcoma recurrence or metastasis, or were alive at the time of data acquisition.

## 3 Results

Sixty-six cats were included for statistical analysis who received bleomycin only ECT for treatment of their sarcomas. Twenty-seven were female spayed, whereas 39 were male castrated. Breeds included one American Longhair, three American Shorthair, six Domestic Longhair, four Domestic Mediumhair, 33 Domestic Shorthair, one Himalayan, five Maine Coons, one Norwegian Forest cat, one Ragdoll, three Russian Blues, two Siamese, three Siamese mixes, and three Tabby cats. Average age of cats was 9.5 years (range: 1.98–19.0 years).

The high-grade group included 43 cats with the following diagnoses provided by veterinary pathologists: two anaplastic sarcomas, one chondrosarcoma, one extraskeletal osteosarcoma, four fibrosarcomas, eight high grade fibrosarcomas, 15 injection site sarcomas, one peripheral nerve sheath tumor, three sarcomas, and eight soft tissue sarcomas with mitotic counts ranging from 10 to 80–100. Tumor margins included 23 incompletely excised sarcomas and 20 marginally excised sarcomas.

The treatment protocol for the high-grade group consisted of two electrochemotherapy sessions; however, four cats received a single session of electrochemotherapy, and one cat received three electrochemotherapy sessions based on owner preference. The first electrochemotherapy session, for this group, was performed at a mean of 35 days postoperatively. The median DFI for this group was 389 days, and the mean DFI was 1,107 days (CI 764–1,450 days). Local recurrence was observed in 46.5% (20/43) of cats, whereas 41.8% (18/43) of cats did not have recurrence at the time of their death or data acquisition. Confirmed or suspected metastasis occurred in 11.6% (5/43) of cats with one cat developing pleural effusion, one cat with lymph node metastasis, one cat with multiple cutaneous and subcutaneous sarcoma lesions along the neck and thorax, one cat with pulmonary nodules, and one cat with sarcoma metastasis to a digit.

ECT-related side effects occurred in 27.9% (12/43) of cats in this group including one grade 1 pain, two grade 2 pain, one grade 1 soft tissue necrosis, three grade 2 soft tissue necrosis, one grade 1 bruising, two grade 2 pruritis, and one grade 1 altered appetite. Following ECT, 23.2% (10/43) of cats received maximum tolerated dose (MTD) chemotherapy with five cats receiving lomustine, two cats receiving doxorubicin, two cats receiving chlorambucil, and one cat receiving a combination of lomustine and doxorubicin. Lomustine was administered to one cat prior to hemipelvectomy and first ECT session. Two cats received the Torigen vaccine (Torigen Pharmaceuticals, Farmington, CT, USA) immunotherapy following electrochemotherapy.

The low-grade sarcoma group included 23 cats with the following diagnoses provided by veterinary pathologists: five cutaneous hemangiosarcomas, two fibrosarcomas, one neurofibrosarcoma, three sarcomas, 11 soft tissue sarcomas, and one spindle cell sarcoma. Location of the low-grade tumors included nine head or neck tumors, six tumors at or below the elbow or stifle, five hip/thigh tumors, one interscapular tumor, one tumor along the shoulder, one tumor along the sternum, and one interscapular tumor. Tumor margins within this group consisted of 18 incompletely excised sarcomas and five marginally excised sarcomas.

The treatment protocol for the low-grade sarcoma group consisted of a single ECT treatment performed at a mean of 79 days postoperatively. The median DFI for this group was not reached, and the mean was 2,658 days (CI 2076–3,239 days). Among the 23 cats, local recurrence occurred in 17.3% (4/23) of cats, while 82.6% (19/23) of cats did not have recurrence at the time of their death or data acquisition. No cats in this group experienced metastasis. ECT-related side effects occurred in 8.6% (3/23) of cats including one grade 1 pain and two grade 2 pruritus. Only one cat in this group received chemotherapy following ECT; chlorambucil was administered for concurrent small cell gastrointestinal lymphoma and no cats received chemotherapy prior to ECT.

Median DFI for low-grade sarcomas described in this study (median days not reached) was significantly (*p* ≤ 0.001) longer than that of high-grade tumors (389 days; Figure 1).

**Figure 1:**
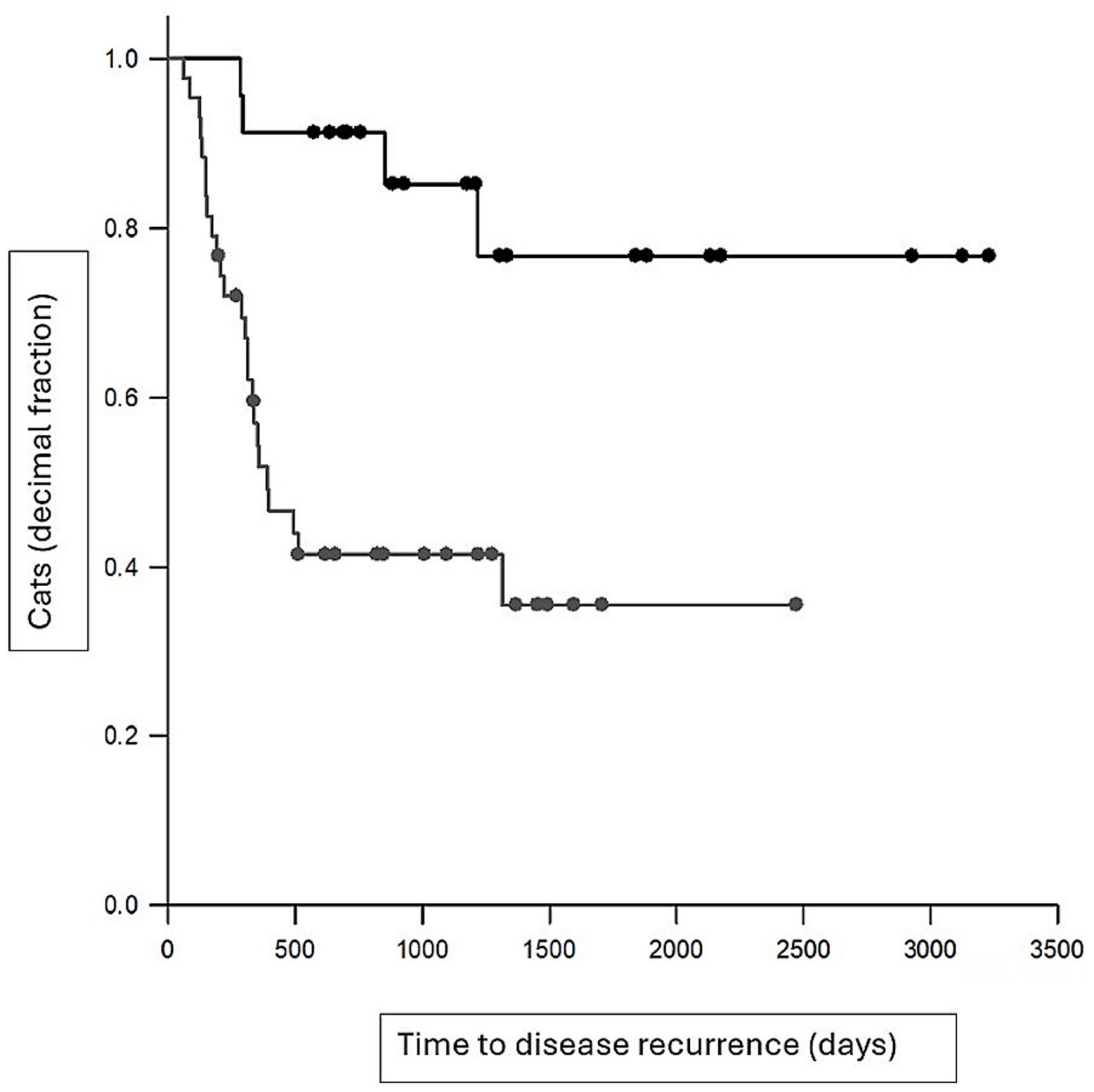
Time-to-event analysis plot: disease-free interval of cats with incompletely or marginally excised high-grade sarcomas (grey) compared to cats with low-grade incompletely or marginally excised sarcomas (black) treated with bleomycin ECT.

Additionally, this study showed that ECT provided limited tumor control to cats with gross disease at the time of ECT. Six cats with gross tumor recurrence were treated with a bleomycin-only electrochemotherapy protocol. Recurrence was confirmed by cytology in one cat, while the remaining five cats were diagnosed based on clinical suspicion arising from physical examination findings. All six cats had rapid progression of their local disease despite multiple ECT treatments. They subsequently underwent debulking/revisional surgery (1 cat) and/or chemotherapy (6 cats; doxorubicin or lomustine) following ECT, with survival times ranging from 102 to 267 days.

## 4 Discussion

Surgery is often considered the primary treatment modality for feline sarcomas (5, 38); however, the adequacy of surgical margins plays a critical role in local tumor control and the risk of recurrence. In a previous study of feline injection-site sarcomas, radical surgical excision and wide surgical excision were associated with a median time to first recurrence (TFR) of 325 days and 419 days, respectively (39). Radical excision was defined as amputation, hemipelvectomy, removal of dorsal spinous processes, and partial scapulectomy whereas wide excision was defined as margins >3 cm (39). This was compared to a TFR of 79 days for tumors treated with marginal excision which was defined as margins <3 cm (39). Cats also benefit from a longer median DFI when the first surgery is performed at a referral institution (39). Given the anatomical constraints that often limit the ability to achieve wide or radical surgical margins, as well as owner preference to avoid more extensive surgery, investigation of adjunctive therapies aimed at delaying or preventing local tumor recurrence is warranted, especially for tumors removed marginally.

Both neoadjuvant and adjuvant radiation therapy have been employed with variable success for incompletely or marginally excised feline injection-site sarcomas. In a more recent study evaluating neoadjuvant radiation therapy to cats with incompletely and completely excised ISSs, the median disease-free interval of cats with incompletely excised ISSs treated with neoadjuvant radiation therapy was 292 days (40). Cats with soft tissue sarcomas treated with neoadjuvant or adjuvant radiation therapy had a median survival of 310 days and 705 days respectively, although surgical margins were not provided (10). Median progression free interval was reported as 37 months for cats treated with adjuvant curative intent radiation therapy (7). Studies evaluating doxorubicin or other chemotherapies (41) in combination with adjuvant radiation therapy have produced mixed results regarding tumor control (8, 9).

There are multiple original studies describing the effects of intravenous bleomycin and intralesional cisplatin ECT on cats with injection site sarcomas (29, 33, 42). Intravenous cisplatin is contraindicated in cats because it induces a fatal pulmonary toxicity (43, 44). To our knowledge, this is the first study to investigate a combined intravenous and intralesional bleomycin electrochemotherapy protocol for the postoperative management of incompletely or marginally excised feline sarcomas divided into high-grade and biologically low-grade groups based on mitotic count alone. Furthermore, this is the first study to evaluate adjuvant treatment for incompletely or marginally excised biologically low-grade feline sarcomas, based on mitotic count alone.

In this study, the median DFI for high-grade tumors was 389 days compared to low-grade sarcomas where the median DFI was not reached. Most of the cats underwent surgical excision of their tumors at their referring veterinarian prior to oncologic consultation. This likely led to delay in first ECT treatment and subsequently shorter median DFI when compared to one of the historic studies evaluating the timing of ECT post-surgical excision for feline injection-site sarcomas (33). In the comparative study referenced, cats were treated with an intralesional bleomycin electrochemotherapy protocol at the time of surgery or 2 weeks post-operatively. The cats treated with ECT intraoperatively had a median time to recurrence of 12 months whereas the cats treated 2 weeks postoperatively had a median time to recurrence of 19 months (33).

Adverse effects were observed in 22% (15/66) of all cats and were generally mild and self-limiting. For patients receiving 2 or more ECT treatments, no ECT dose reductions were required.

Limitations of this study include its retrospective design and conduct at a single institution. However, the use of a standardized treatment protocol across all cases provided consistency in patient management and was associated with favorable clinical outcomes over an extended period.

Pathologic grading of sarcomas in cats still lacks solid guidelines that are consistently used. This variability creates diagnostic discordance in discussing risk with owners. Recognizing the potential for tumor recurrence, many owners opted for additional therapy to maximize the duration of local tumor control. The relatively low number of recommended ECT sessions, minimal associated adverse effects, and limited availability of radiation therapy facilities made ECT an attractive treatment option.

Outcomes and overall DFI were evaluated through physical exam. Not all cases of tumor recurrence were confirmed cytologically or histologically, which may have affected outcome assessment.

Moving forward, a prospective study comparing intralesionally administered bleomycin to intralesionally administered cisplatin during ECT treatments would be ideal to determine if there is an advantage to intralesional cisplatin for cats treated with electrochemotherapy.

ECT was well-tolerated in this study, which is a repeatable finding amongst all studies evaluating this therapy in cats. The mean DFI of both sarcoma groups exceeding 1,000 days further supports ECT’s potential as an effective post-operative alternative for oncologic practices without access to radiation therapy.

## Data Availability

The original contributions presented in the study are included in the article/supplementary material, further inquiries can be directed to the corresponding author/s.
